# Effectiveness of clinical training on improving essential newborn care practices in Bossaso, Somalia: a pre and postintervention study

**DOI:** 10.1186/s12887-020-02120-x

**Published:** 2020-05-13

**Authors:** Ribka Amsalu, Catherine N. Morris, Michelle Hynes, Hussein Jama Had, Joseph Adive Seriki, Kate Meehan, Stephen Ayella, Sammy O. Barasa, Alexia Couture, Anna Myers, Binyam Gebru

**Affiliations:** 1 Department of Global Health, Save the Children, Washington, DC 20002 USA; 2 Center for Global Health. US Centers for Disease Control and Prevention, 1600 Clifton Rd, Atlanta, GA 30329-4027 USA; 3 Save the Children International, Mogadishu, Somalia; 4 Kenya Medical Training College, Chuka Campus, Nairobi, Kenya; 5 Independent consultant, New York, NY USA

**Keywords:** Essential newborn care, Humanitarian emergencies, Conflict, Clinical training, Somalia

## Abstract

**Background:**

Increasingly, neonatal mortality is concentrated in settings of conflict and political instability. To promote evidence-based practices, an interagency collaboration developed the Newborn Health in Humanitarian Settings: Field Guide. The essential newborn care component of the Field Guide was operationalized with the use of an intervention package encompassing the training of health workers, newborn kit provisions and the installation of a newborn register.

**Methods:**

We conducted a quasi-experimental prepost study to test the effectiveness of the intervention package on the composite outcome of essential newborn care from August 2016 to December 2018 in Bossaso, Somalia. Data from the observation of essential newborn care practices, evaluation of providers’ knowledge and skills, postnatal interviews, and qualitative information were analyzed. Differences in two-proportion z-tests were used to estimate change in essential newborn care practices. A generalized estimating equation was applied to account for clustering of practice at the health facility level.

**Results:**

Among the 690 pregnant women in labor who sought care at the health facilities, 89.9% (*n* = 620) were eligible for inclusion, 84.7% (*n* = 525) were enrolled, and newborn outcomes were ascertained in 79.8% (*n* = 419). Providers’ knowledge improved from pre to posttraining, with a mean difference in score of + 11.9% (95% CI: 7.2, 16.6, *p*-value < 0.001) and from posttraining to 18-months after training with a mean difference of + 10.9% (95% CI: 4.7, 17.0, p-value < 0.001). The proportion of newborns who received two or more essential newborn care practices (skin-to-skin contact, early breastfeeding, and dry cord care) improved from 19.9% (95% CI: 4.9, 39.7) to 94.7% (95% CI: 87.7, 100.0). In the adjusted model that accounted for clustering at health facilities, the odds of receiving two or more essential newborn practices was 64.5 (95% CI: 15.8, 262.6, *p*-value < 0.001) postintervention compared to preintervention. Predischarge education offered to mothers on breastfeeding 16.5% (95% CI: 11.8, 21.1) vs 44.2% (95% CI: 38.2, 50.3) and newborn illness danger signs 9.1% (95% CI: 5.4, 12.7) vs 5.0% (95% CI: 2.4, 7.7) remained suboptimal.

**Conclusions:**

The intervention package was feasible and effective in improving essential newborn care. Knowledge and skills gained after training were mostly retained at the 18-month follow-up.

## Background

Increasingly, neonatal mortality and stillbirth are concentrated in settings of conflict and political instability [1]. Four of the five countries with the highest neonatal mortality rates in the world are in a state of chronic conflict or political instability: Somalia, South Sudan, Afghanistan and Pakistan [2]. Despite this burden, insufficient information is available on effective newborn health implementation approaches in humanitarian emergencies [3]. While there are global strategies and guidelines on newborn health for resource-poor and high mortality settings, strategies on how to scale-up evidence-based newborn interventions in the context of conflict and humanitarian emergency are lacking [3]. To promote evidence-based practices and provide guidance on neonatal care in humanitarian emergencies, an interagency working group developed the Newborn Health in Humanitarian Settings: Field Guide (Field Guide) [4]. The Field Guide, rooted in evidence-based practices recommended by the World Health Organization (WHO), comprises lists of interventions, neonatal medical supplies and drugs, and monitoring approaches at the community, primary health care, and hospital levels. In this study, we applied the essential newborn care included in the primary health facilities section of the Field Guide.

Much of the effect of conflict and disaster is experienced by communities that reside outside formal camps where access to quality health services is limited [3, 5]. In settings such as Somalia, childbirth often occurs at home or at the lowest level of the health system [6]. As neonatal mortality risk peaks at birth and during the first 24 h of life [7], it is critical to test the feasibility and to generate an evidence base for how and what can be implemented at the primary level by mid-level health workers (nurses and midwives) close to the community to improve newborn survival. Earlier studies in developing countries have shown that introducing a tailored package of essential newborn care practices might reduce stillbirth and newborn mortality [8, 9]. WHO defines essential newborn care (ENC) as a set of interventions and practices provided at childbirth and immediately after birth that includes thermal care, hygienic practices during childbirth, early breastfeeding, and newborn resuscitation [10]. As these essential newborn care practices need to be provided during labor, birth and immediately after birth, it is critical that health workers who are responsible for service provision in the labor/maternity unit have the knowledge and skills and the medical supplies necessary to provide safe and timely care.

There are two commonly used training curricula designed to build the knowledge and skills of health workers in essential newborn care: the WHO’s Essential Newborn Care Course and the American Academy of Pediatrics (AAP) Helping Babies Survive (HBS) program [11, 12]. The WHO and AAP training courses have varied levels of depth, duration, and capacity building approaches. While trainings based on the WHO and AAP training curricula have shown improvement in providers’ knowledge and skills immediately after training, their effects on changes in clinical practice, newborn mortality and stillbirth are inconsistent [13–16]. We applied the AAP HBS curriculum, since it contained substantive practical sessions for improving the skills of providers, and we supplemented the curriculum with intrapartum and maternal modules as recommended by the Field Guide.

We conducted the essential newborn care feasibility and effectiveness study in Somalia, a country that has experienced more than three decades of armed conflict [17]. At the national level, health indicators in Somalia are poor, with a maternal mortality ratio of 829 per 100,000 live births in 2017, and an estimated neonatal mortality rate of 38 deaths per 1000 live births in 2018 [2, 18]. The main causes of neonatal death in 2015 were birth asphyxia and trauma, 38.6%; prematurity, 21.1%; and infections, 28.3% [19]. The 2011 Multi Indicator Cluster Survey (MICS) in Puntland, the autonomous region of Somalia where this study was conducted, showed low antenatal coverage at 27%, low institutional delivery at 13%, and low early initiation of breastfeeding at 56% [6].

We performed a prepost intervention study to determine whether the essential newborn care practices recommended in the Field Guide were feasible to implement at the primary facility level and to measure the effect of the intervention package on increasing the correct and timely use of essential newborn care practices including: (1) thermal care, (2) breastfeeding, (3) hygienic childbirth practices, and (4) newborn resuscitation. The intervention package comprised training of health workers, provision of newborn medical supplies and drugs, and installing of newborn data collection systems. We hypothesized that the intervention package would improve essential newborn care practices by 15% from an estimated baseline (preintervention) prevalence of 30%.

## Methods

### Study design and timeline

This quasi-experimental prepost intervention study was conducted in Bossaso, Somalia from August 2016 through December 2018. Mixed data collection procedures included health worker knowledge and skills evaluations; observations of essential newborn care practices during labor, birth, and immediately after birth; postnatal interviews of mothers at home or via phone on the 7th -9th day after birth; and in-depth interviews and focus group discussions with health workers.

**Fig. 1:**
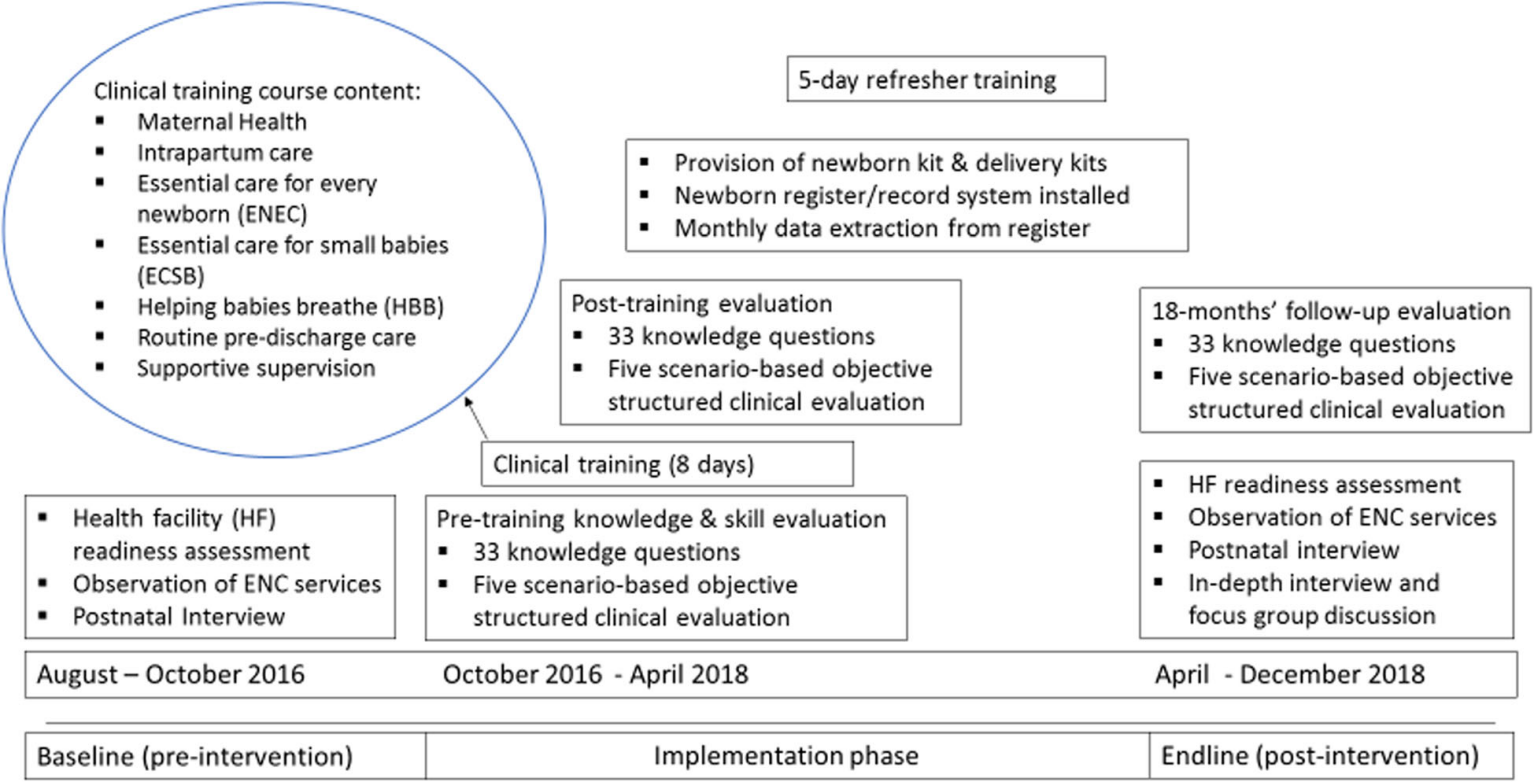
Schematic overview of essential newborn care Package implementation

Preintervention baseline measurements were recorded from August to October 2016. An intervention package consisting of the clinical training for health workers, provision of newborn medical kits, and installation of a newborn health record system was implemented from October 2016 to April 2018. The postintervention endline measurements were taken from April to December 2018 (Fig. 1).

### Study settings & study participants

In Somalia, the health system has four levels: hospitals, referral health centers, health centers, and health posts [20]. Health centers are also known as maternal and child health centers. In Bossaso city, six health centers and one public hospital provide maternal and child services to internally displaced persons (IDPs) and to the host community. In consultation with the Ministry of Health, we selected four of the six health centers serving IDPs based on predefined selection criteria: the health facilities were open 24 h a day, 7 days a week and had an average of at least 40 deliveries per month. The total catchment population of the four health facilities was estimated at 134,735 persons, including both the IDPs and the host community.

Pregnant women 15–49 years of age who sought childbirth care at one of the study facilities during the study period were eligible and approached for consent and enrollment in the study. Women who were immediately referred to a hospital prior to childbirth were excluded. Women who had a stillbirth or early newborn death defined as death from 0 to 7 days of life were excluded from the postnatal interview out of respect for the family. Health workers who were responsible for service provision in the labor/maternity unit and the in-charges at the four health facilities were approached for consent and included in the study.

### Implementation of newborn intervention package

The successful translation of the Field Guide to Practice necessitates the training of health workers in essential newborn care practices, the provision of newborn medical supplies and drugs, and the installation of data collection systems. The curriculum for the training of health workers was based on the AAP HBS program and comprised the Helping Babies Breathe (HBB), Essential Care for Every Baby (ECEB), and Essential Care for Small Babies (ECSB) [12]. Supplemental modules on maternal health, intrapartum care, identification and management of maternal complications, and supportive supervision were also included in the 8-day course. The training was taught by two experienced clinicians from Kenya with expertise in the subject area and experience as educators. The 8-day essential newborn care course was taught through various teaching methods: didactic lectures, videos developed for educational purposes, small group discussions, and skills practice with NeoNatalie, MamaBreast simulator, and a partograph [21–24]. Skills practices were performed in pairs and covered the assessment of a newborn, immediate newborn care, newborn resuscitation, and completion of a partograph on simulated case stories. The trainees were midwives, registered nurses, and in-charges at the four health centers. A 5-day refresher training was conducted 6 months after the initial course focusing on review of the topics covered during the initial training. Knowledge and skills evaluations were conducted pretraining, immediately after training (posttraining), and at an 18-month follow-up.

Twelve health workers (three per health center) were trained, representing 43–46% of all registered nurses and midwives in the four health facilities. Overall, there were 26 registered nurses or midwives working at the four health facilities at baseline, and 28 registered nurses or midwives at endline. We were unable to train all the health workers due to funding limitations and the requirement for some providers to remain on duty.

Newborn kits and delivery kits containing the medical supplies and drugs recommended by the Field Guide for the primary health facility level were distributed to the four health centers. The kits included the supplies and medicines necessary to monitor labor, attend childbirth, perform newborn examinations, perform newborn resuscitation, provide routine predischarge care, antibiotics, and medications for maternal health (Supplement 1: List of medical supplies and drugs)*.*

As the existing labor and delivery registers lacked key information on the newborns, a supplemental newborn register was installed. The newborn register included information on gestational age, date and time of birth, birth weight, and any newborn complications observed (Supplement 2: Newborn register book*).* The newborn register was created in English and translated into Somali. The Somali version of the register was printed and distributed to the health facilities; health workers were trained and received orientation on how to complete the register book and extract data monthly.

### Study outcomes and data collection

The primary outcome was a composite indicator of the essential newborn care practices and services provided to newborns at birth and immediately after birth and was measured via direct observation of clinical practices using an observation checklist. The observation checklist was adapted from the WHO’s Managing Complications in Pregnancy and Childbirth Guide [25], which has been validated in African and conflict settings [26, 27]. The observers were female from the community and with a health background (midwifery and nursing students). All observers received didactic and video-based simulation training that demonstrated the recommended essential newborn care practices. The observation tool was piloted for 2 days during the preintervention baseline assessment by pairing observers. Interobserver agreement was 94.6%.^1^ The primary outcome, essential newborn care, was defined as the proportion of newborns that were observed to receive at least two essential newborn care interventions. Additional variables measured were care received during pregnancy (history), predischarge education given to the mother, and maternal and newborn outcomes. Postnatal interviews with mothers were conducted either by phone or in person on the 7th to 9th day after birth. The postnatal interviews captured the status of the newborn, the mother’s knowledge of danger signs and newborn care practices, and the mother’s level of satisfaction with the care received at the health facility.

Scores of the health worker knowledge test and skills evaluation were collected for all training participants*.* Twelve health workers participated in the training and were evaluated at pretraining, posttraining, and at the 18-month follow-up. The knowledge and skill evaluation tools, the multiple-choice questionnaire (MCQ) and scenario-based Objective Structured Clinical Examinations (OSCE), were adapted from the AAP course. The lifesaving skills emergency obstetric training case studies on partograph use were applied to evaluate skills in the accurate completion and use of the partograph [24].

Qualitative assessment was performed at the endline. Three Focus Group Discussions (FGDs) were held with 30 health workers at the four health centers (one with providers who received the training, two with providers who had not), and in-depth interviews (IDIs) were conducted with four in-charges. The qualitative assessment aimed to examine whether and how the 8-day course and refresher training were shared among health workers who had not participated in the training; to gather information from those who attended the training on what had changed in their clinical practice after course completion; to gain insight into the perspectives of the health workers (trained and untrained) on the utility of the newborn commodities and medical supplies; and to collect their feedback on the installed newborn record/register system. The FGDs and IDIs were either conducted in English with a Somali interpreter or in Somali and audio recorded then translated and transcribed into English.

### Sample size

We assumed a baseline prevalence of the provision of essential newborn care of 30%, a power of 80%, a 5% probability of a Type I error, and a nonresponse rate of 20%. To detect an absolute difference in the primary outcome, provision of essential newborn care, of at least 15%, the number of mother-newborn pairs needed was 203 at both pre and postintervention. The sample size for each facility was allocated using a proportional to estimated size where the size measure was based on historic data on number of childbirths per health facility [28].

### Statistical analysis

Descriptive statistics were used to summarize the data. Proportions with 95% CI, mean (standard deviation), and median (interquartile range) were generated to characterize the study population. Missing and “don’t know” responses were analyzed as missing and excluded from the analysis. To estimate the effect of the training on the trainees’ knowledge and skills, paired Student’s t-tests were used. Differences in two proportion z-tests were used to test for differences in proportions in maternal and newborn characteristics and essential newborn care practices pre and postintervention. To accommodate for possible correlations of changes in score with health facility, i.e., single level of clustering at the health facility level, we ran a generalized estimating equation (GEE) with robust option. The GEE utilized health facility as the cluster, the indicators of interest as an outcome, and the preintervention versus postintervention indicator as predictor to estimate the adjusted odds ratio, F-statistic and *p*-value. We applied logistic regression to estimate the risk ratio, an approximation of the odds ratio for rare events, of early neonatal mortality and stillbirth. Statistical significance was considered at a *p*-value < 0.05. STATA (StataCorp. 2015. *Stata Statistical Software: Release 14*. College Station, TX: StataCorp LP) was used for the quantitative analysis. For the qualitative analysis, audio recordings from the IDIs and FGDs were transcribed into English and imported into MAXQDA Analytics Pro (VERBI Software, 2017) for coding and analysis. Researchers engaged in content analysis applying an initial coding list based on the interview and focus group discussion guides. New codes were added as they emerged during the analysis. Once the coding framework was finalized, a subset of transcripts was coded by a primary and a secondary analyst to determine the intercoder reliability. The themes that emerged were consolidated into four subthemes.

### Ethical compliance

Approval for the study was sought from the Puntland Ministry of Health, Save the Children and the Centers for Disease Control and Prevention (CDC). Approval was obtained from the Puntland Ministry of Health, the Save the Children ethics review committee, and a nonresearch determination approved by the CDC. Consent information was read to the women in the local language, and verbal consent was sought at each point of contact. Those who consented were included in the study. Personal identifiers collected to facilitate postnatal follow-up visits were destroyed immediately after completion of the data collection process. Verbal consent was obtained from health workers and the in-charges who were responsible for service provision at the four health facilities.

## Results

**Fig. 2:**
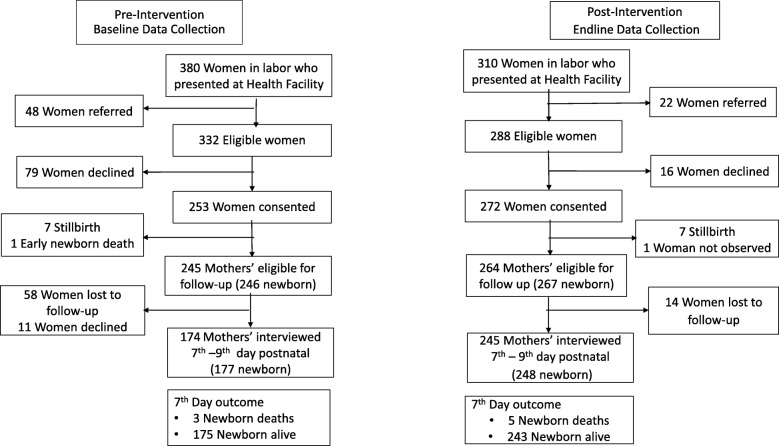
Study flow chart

Overall, 690 pregnant women in labor sought care at the four health centers; 89.9% (*n* = 620) were eligible, 84.7% (*n* = 525) consented and were enrolled in the study, and outcomes were ascertained in a postnatal follow-up assessment in 79.8% (*n* = 419) of enrolled women (Fig. 2).

On average, there were 1 to 2 births per day per health facility, and the highest proportion of births occurring at health center 2 at both baseline and endline. Birth attendants at the health facilities were either community midwives, auxiliary nurses, registered nurses or midwives. A minimum of three registered nurses and three midwives were available per facility at baseline; no registered nurses and 3 midwives were available at endline. At study baseline and endline, the proportion of births attended by a midwife was 90.5% [95% CI: 86.2, 93.8] and 89.3% [95% CI: 85.0, 92.7], respectively. The pre and postintervention obstetric history of the women who presented in labor with regard to median maternal age [interquartile range] (25 yrs. [21, 29] vs 26 yrs. [22, 29]), primigravida (19.8% [95% CI: 15.0, 25.2] vs 14.4% [95% CI: 10.4, 19.1]), and at least one antenatal care visit (85.3% [95% CI: 75.3, 95.7] vs 88.2% [95% CI: 78.3, 98.4]) were comparable (Table 1). The proportion of newborns born preterm were comparable at pre and postintervention. There was variation in the proportion of newborns born with low birth weight with an increase at postintervention measurement (2.5% [95% CI: 0.7, 5.3] vs 7.9% [95% CI: 4.2, 10.7]) (Table 2).

**Table 1:** Health facility and study participant characteristics

	Pre-intervention			Post-intervention			*P*-value
	n/N	%	95% CI	n/N	%	95% CI	
Facility characteristics							
Maternity/labor room functional 24/7	4/4	100		4/4	100	-	-
Electricity functional	3/4	75		2/4	50		0.57
Water availability	4/4	100		3/4	75		0.29
Health worker							
Mean skilled birth attendants per facility (registered nurse)	3.8	Range (3–6)		2.8	Range (0–4)		
Mean skilled birth attendants per facility (midwife)	3.5	Range (3–4)		3.5	Range (3–5)		
Location of birth							
Health center 1	55/253	21.7	16.8–27.3	75/271	27.7	22.4–33.4	0.44
Health center 2	95/253	37.5	31.6–43.8	93/271	34.3	28.7–40.3	0.64
Health center 3	58/253	22.9	17.9–28.6	60/271	22.1	17.3–27.5	0.92
Health center 4	45/253	17.8	13.3–23.1	43/271	15.9	11.7–20.8	0.81
Length of stay from birth to discharge at HF among livebirths							
≤ 6:00 h	161/242	66.5	60.6–72.4	174/257	67.7	62.0–73.4	–
6:00–12:00 h	61/242	25.2	19.7–30.7	67/257	26.1	20.7–31.5	–
≥ 12:01 h	20/242	8.3	4.8–11.7	16/257	6.2	3.3–9.2	–
Birth attendant							
Midwife	229/253	90.5	86.2–93.8	242/271	89.3	85.0–92.7	0.66
Registered nurse	13/253	5.1	2.8–8.6	3/271	1.1	0.2–3.2	0.58
Auxiliary nurse	9/253	3.6	1.6–6.6	23/271	8.5	5.4–12.5	0.62
Community midwife	2/253	0.8	0.1–2.8	3/271	1.1	0.2–3.2	0.97
Maternal age							
15–18 years	23/253	9.1	5.9–13.3	16/271	5.9	3.4–9.4	0.20
19–24 years	78/253	30.8	25.2–36.9	88/271	32.5	26.9–38.4	0.77
≥ 25 years	152/253	60.1	53.8–66.2	167/271	61.6	55.5–67.4	0.86
Gravidity							
Primigravida	50/253	19.8	15.0–25.2	39/271	14.4	10.4–19.1	0.17
Two or more pregnancies	203/253	80.2	74.8–85.0	232/271	85.6	80.9–89.6	0.62
Antenatal care during this pregnancy							
None	31/253	12.2	8.5–16.9	32/271	11.8	8.2–16.2	0.96
One to three ANC visits	181/253	71.5	65.5–77.0	196/271	72.3	66.6–77.6	0.87
Four or more ANC visits	35/253	13.8	9.8–18.7	43/271	15.9	11.7–20.8	0.80
Missing values	6/253	–		0/271	–		–

**Table 2:** Maternal and newborn birth outcomes and complications

Variable	Pre-intervention			Post-intervention			*P*-value
	n/N	%	95% CI	n/N	%	95% CI	
Birth and newborn outcome							
Live birth (Singleton)	245/253	96.8	93.9–98.6	261/271	96.3	93.3–98.2	0.96
Live birth (Twins)	1 /253	0.4	0.01–2.2	3 /271	1.1	0.2–3.2	0.34
Estimated gestational age							
< 37 Weeks	6/247	2.4	0.9–5.1	4/267	1.5	0.4–3.7	0.45
≥ 37 Weeks	240/247	97.2	94.4–98.9	263/267	98.5	96.3–99.6	0.91
Missing values	1/247	–		–	–		
Birthweight							
< 2500 g	6/247	2.5	0.7–5.3	21/267	7.9	4.2–10.7	0.002
≥ 2500 g	216/247	87.4	94.7–99.3	245/267	91.8	89.3–95.8	0.71
Missing values	25/247	10.1	6.7–14.6	1	0.4	0.0–2.1	< 0.001
Newborn complications							
Severe infection	5 /247	2.0	0.7–4.7	7 /267	2.6	1.1–5.3	0.66
Isolated fast breathing	4 /247	1.6	0.4–4.1	1 /267	0.4	0.0–2.1	0.15
Birth asphyxia	11 /247	4.5	2.2–7.8	13 /267	4.9	2.6–8.2	0.83
Congenital abnormality	0 /247	0.0		1 /267	0.4	0.0–2.1	0.34
Other	2 /247	0.8	0.1–2.9	1 /267	0.4	0.0–2.1	0.52
Maternal complications^a^							
Bleeding	16 /253	6.3	3.7–10.1	7 /271	2.6	1.0–5.2	0.05
Obstructed/prolonged labor	17 /253	6.7	4.0–10.5	3 /271	1.1	0.2–3.2	0.001
Pre-eclampsia/eclampsia	4 /253	1.6	0.4–4.0	0 /271	0.0		0.04
Severe infection	12 /253	4.7	2.5–8.1	12/271	4.4	2.3–7.6	0.87
Other	3 /253	1.2	0.2–3.4	0 /271	0.0		0.07

^a^zero maternal death was reported at pre- and post-intervention measurement

### Health worker knowledge and skills

**Fig. 3:**
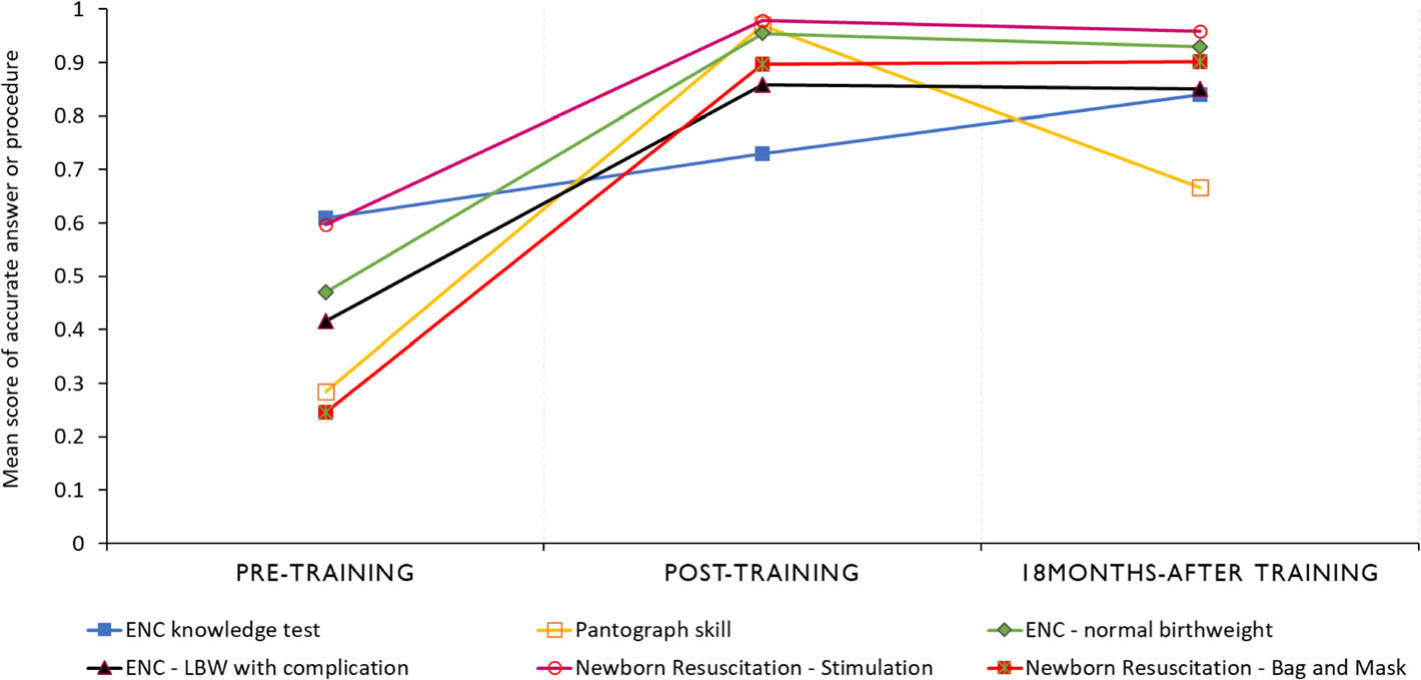
Mean score of knowledge and skills evaluation: answers and procedures performed accurately by health providers. ENC knowledge score was based on a 33-item multiple-choice questionnaire

All 12 registered nurses and midwives who attended the training completed the multiple-choice knowledge test questionnaire and the scenario-based objective structured clinical skill evaluation at three time points. Their knowledge scores improved from pre to posttraining and at the 18-month follow-up. The mean difference in score from posttraining to pretraining was + 11.9% [95% CI: 7.2, 16.6; *p*-value < 0.001] and from 18-months after training to posttraining was + 10.9% [95% CI: 4.7, 17.0; *p*-value < 0.001] (Fig. 3). The score on the accurate completion of a partograph at baseline was 28.5% (95% CI: 11.7, 45.3), which improved at posttraining with a mean difference in score of + 68.5% (95% CI: 52.7, 84.3; *p*-value < 0.001) and declined at the 18 month follow-up from posttraining with a mean difference in score of − 30.3% (95% CI: − 13.5, − 47.1; *p*-value = 0.002). Skills in newborn resuscitation with bag and mask improved from pretraining to posttraining with a mean difference in score of + 65.1% (95% CI: 53.4, 76.7; *p*-value < 0.001), and the skill was retained at the 18-month follow-up from the posttraining score with a mean score difference of + 0.4 (95% CI: − 6.6, 7.4; *p*-value = 0.903) (Fig. 3).

### Essential newborn care practices

**Fig. 4:**
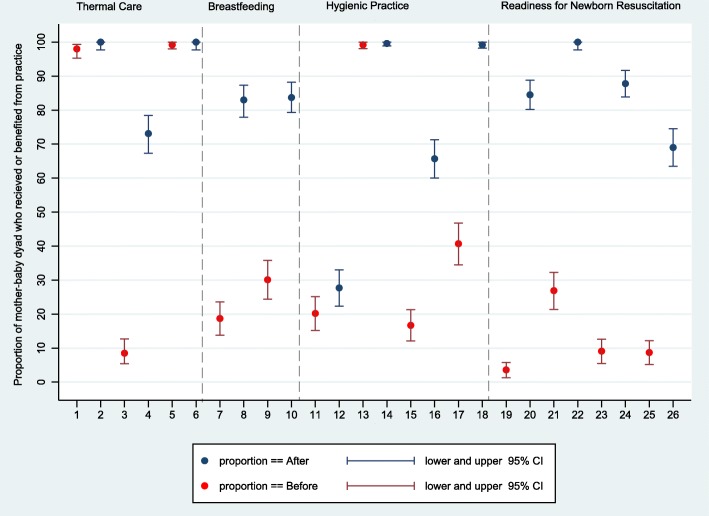
Observed change in essential newborn care practice readiness and care provided to mother-baby dyad. X-axis label 1,2 = immediate drying; 3,4 = skin-to-skin contact; 5,6 = delayed bathing; 7,8 = support in initiation of breastfeeding; 9,10 = early breastfeeding; 11,12 = provider washes hands with soap & water; 13,14 = provider wears new sterile gloves; 15,16 = provider uses sterile or clean delivery kits; 17,18 = dry cord care; 19,20 = printed partograph in labor room; 21,22 = functioning fetoscope in labor room; 23,24 = resuscitation surface/table in labor room; 25,26 = bag/mask in labor room

The primary composite outcome, proportion of newborns who received two or more essential newborn care practices (skin-to-skin contact, early breastfeeding, and dry cord care), improved from 19.9% (95% CI: 4.9, 39.7) at baseline to 94.7% (95% CI: 87.7, 100.0) at endline with a difference in proportion of + 74.8% (95% CI: 69.1, 80.5; *p*-value < 0.001). In the adjusted model, the odds of receiving two or three newborn practices at endline versus baseline was 64.5 (95% CI: 15.8, 262.6, *p*-value < 0.001). The proportion of newborns who received three newborn care practices improved from 0.8% (95% CI: 0.0, 1.7) at baseline to 61.4% (95% CI: 37.8, 77.0) at endline, with a difference in proportion of + 60.6% (95% CI: 54.6, 66.5; *p*-value < 0.001) (Table 3). All newborns who had birth asphyxia were successfully resuscitated both pre and postintervention. The difference in the proportion of newborns who received skin-to-skin contact was + 64.6% (95% CI: 58.2, 71.0, *p*-value < 0.001) from a baseline of 8.5% (95% CI: 5.4, 12.7) and in early breastfeeding was + 53.6% (95% CI: 46.4, 60.9, *p*-value< 0.001) from a baseline of 30.1% (95% CI: 24.4, 35.8) (Fig. 4).

**Table 3:** Observed essential newborn care practices adjusted for health facility

	Pre-Intervention		Post-Intervention		Difference	Adjusted Odds Ratio (GEE)	***p***-value (GEE)
	n/N	% (95% CI)	n/N	% (95% CI)	% (95% CI)	% (95% CI)	
Early initiation of breastfeeding	74/246	30.1(8.8, 61.4)	221/264	83.7(59.4, 98.6)	53.6(46.4, 60.9)	10.6 (1.6, 69.8)	0.014
Thermal care^a^	21/246	8.5 (0.0, 21.3)	190/264	72.0 (59.4, 86.1)	63.4 (57.0, 69.9)	28.4 (8.0, 100.9)	< 0.001
Clean childbirth practices ^b^	6/246	2.4 (0.0, 21.3)	70/264	26.5 (7.9, 46.9)	24.1 (18.4, 29.7)	11.1 (2.6, 46.6)	0.001
Newborns received at least two ENC practices ^c^	49/246	19.9 (4.9, 39.7)	250/264	94.7 (87.7, 100)	74.8 (69.1, 80.5)	64.5 (15.8, 262.6)	< 0.001
Newborns received three ENC practices ^d^	2/246	0.8 (0.0, 1.7)	162/264	61.4 (37.8, 77.0)	60.6 (54.6, 66.5)	220.0 (33.7, 1443.0)	< 0.001
Newborn that needed resuscitation	34/246	13.8 (8.3, 22.0)	16/264	6.1 (4.4, 8.3)	−7.8(−13.3, −2.3)	0.4 (0.2, 1.0)	0.057
Newborns who started breathing after stimulation	23/23	100%	0	0	–	–	
Newborns who started breathing after bag & mask resuscitation	11/11	100%	16/16	100%	–	–	

^a^ Newborn received all three thermal care practices: immediate drying, skin-to-skin contact, delayed bathing while in the facility

^b^ Hygienic childbirth practices all five adhered: visibly clean delivery bed, handwashing of attendant, gloves wore by attendant, use of sterile delivery kit, and dry cord care

^c^ Newborn received two out of these three practices: skin-to-skin contact, early initiation of breastfeeding, dry cord care

^d^ Newborn received all three practices: skin-to-skin contact, early initiation of breastfeeding, dry cord care

**Fig. 5:**
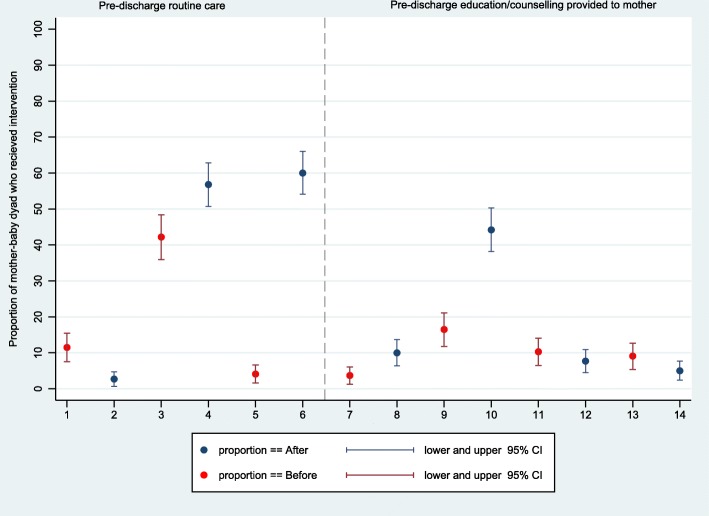
Observed change in essential newborn care practices: predischarge routine care and education provided to mother-baby dyad. X-axis label: 1,2 = examination of newborn 2 h after birth; 3,4 = eye ointment provided; 5,6 = vitamin K provided; 7,8 = education on skin-to-skin contact provided; 9,10 = education on breastfeeding provided; 11,12 = education on dry cord care provided; 13,14 = education on danger signs of newborn illness provided

The differences in essential newborn practices was one directional (improvement) for all health facilities for most of the indicators. However, for predischarge care and education, handwashing by the attendant, and use of the sterile delivery kit, the direction of change varied by health facility. Overall, predischarge education provided to mothers related to newborn care at pre vs postintervention on the topics of skin-to-skin contact (3.7% (95% CI: 1.3, 6.1) vs 10.0% (95% CI: 6.4, 13.7)), breastfeeding (16.5% (95% CI: 11.8, 21.1) vs 44.2% (95% CI: 38.2, 50.3)), and danger signs of newborn illness (9.1% (95% CI: 5.4, 12.7) vs 5.0% (95% CI: 2.4, 7.7)) remained suboptimal (Fig. 5).

This study was not powered to detect changes in mortality. From the data gathered, the proportion of stillbirth was 2.8% (95% CI: 1.1, 5.6) at baseline and 2.6% (95% CI: 1.0, 5.3) at endline, with a risk ratio of 0.9 (95% CI: 0.3, 2.6; *p*-value = 0.899). Early newborn mortality was 1.7% (95% CI: 0.4, 4.9) at baseline and 2.0% (95% CI: 0.7, 4.6) at endline, with a risk ratio of 1.2 (95% CI: 0.3, 4.9, *p*-value = 0.804).

### Health workers’ perspectives on training and knowledge transfer

The themes that emerged from the FGDs and IDIs were summarized in four subthemes: knowledge and skills gained; dissemination of training; applicability of the medical supplies and kits received; and use of the newborn register. In the FGDs and IDIs, health workers reported that the training taught them new skills and improved their previously acquired skills and knowledge.

*“There’s an encouragement for the staff to conduct their services. Even your mind changes – our mind has changed when we got that training and knowledge*.” (In-depth interview participant)

Interventions that resulted in self-reported areas of change included Kangaroo Mother Care (KMC) and recognition of the golden minute for newborn resuscitation. Participants stated that they had been taught about the importance of skin-to-skin contact previously as an intervention for low birth weight babies, but after the study intervention period, they now used skin-to-skin contact for all babies delivered in their facilities.

Notably, changes in care resulting from the intervention were reported in the FGDs with health workers who had not attended the training as well as those who attended the training, most likely due to dissemination efforts within the facilities. All participants from the FGDs and IDIs noted that there was a diffusion of learning within their facilities following the training. Many participants shared that this diffusion of knowledge and materials was an expectation within their facilities. Information was shared through didactic presentations, dissemination of training materials, hands-on demonstrations, on-the-job training, or through a combination of these methods. In some facilities, health workers who had attended the training were paired for multiple shifts with a colleague who had not attended the training.

*“You know some of our staff get trained – they share with other colleagues. That is normal.”* (Focus group discussion participant)

*“Every trained person was assigned to train other untrained staff during shifts, one trained and one untrained in one shift.”* (Focus group discussion participant)

Participants discussed the newborn medical supplies and drugs that the health centers received as part of the study. They commented that some of the medical supplies and drugs in the kit were useful and that they would have liked to have more delivery kits, baby caps, towels, vitamin k, and antibiotics. Some supplies were not used because they either did not know how to use the medical equipment (for example, the vacuum extraction delivery kit) or the supplies were not installed (for example, the table for baby reanimation with overhead heater). The participants also discussed their preference for single use medical supplies, such as disposable newborn suction devices, as they found cleaning and sterilizing instruments challenging.

The participants discussed that the newborn register documents more information on the newborn than they had collected in the past, and since the installation of the newborn register, they have changed the way they record data. For example, newborn birth weight, previously recorded in kilograms, is now recorded in grams. The register also facilitated easier review of information. However, participants voiced challenges associated with the register, they reported that they recorded large amounts of information and that it added to their workload.

## Discussion

This prepost intervention study in a humanitarian emergency demonstrates that an essential newborn care intervention package comprising health worker training, provision of newborn medical commodities, and installation of a newborn register was feasible and associated with a statistically significant improvement in observed essential newborn care practices. There was marked improvement in the proportion of newborns who received two or more essential newborn care practices. In the analysis of health worker knowledge and skills test results, the study found a statistically significant improvement in knowledge and skills immediately posttraining, and the knowledge gained was retained at the 18-month follow-up. Previous studies have shown a reduction in stillbirth after the implementation of essential newborn care training [14]. Our study was not powered to detect changes in stillbirth or early newborn mortality.

Evidence of the applicability of the AAP HBS course in humanitarian emergencies is limited [30]. Our study found that the adapted AAP HBS training curriculum was effective in increasing providers’ knowledge and skills as assessed at posttraining. Critically, our study showed that trained providers retained the knowledge gained at the 18-month follow-up, and skills such as newborn resuscitation with a bag and mask were retained. Studies in Kenya and India that evaluated the retention of knowledge after Helping Babies Breathe training found that knowledge was retained at 6-months’ follow-up; however, a decline in skills in newborn resuscitation with a bag and mask was observed at the 6-month follow-up [16]. Supportive activities, which included a refresher training at the 6th month of implementation, monitoring of the newborn register data throughout the implementation period, and training of health centers’ in-charges in supportive supervision, might have led to the knowledge and skills retention. Similar results were reported in Nepal where continued practice on manikins and coaching was associated with skill retention on newborn resuscitation at the 6-month follow-up [31].

Bundling evidence-based interventions into a service delivery model is more cost-effective than single intervention approaches [8]. In our study, the four areas of essential newborn care practices, including thermal care, early breastfeeding, hygienic birth practices, and readiness to manage birth asphyxia improved after the implementation of the intervention package, providing evidence regarding what is feasible to implement under challenging operational environments. Our study demonstrated that it is possible to train a portion of providers and have the skills transferred to untrained providers through formal and informal methods. The Lancet Neonatal Survival series recommended clinical services be universally provided by skilled health workers to every newborn around the clock [8]. Our study demonstrated that several essential newborn care practices were nearly universal for all newborns attended at endline. While there was variation in the scale of change from pre to-post-intervention, the direction of change improved for most essential newborn practices for all four health centers.

However, care practices such as attendant handwashing with soap and water, though improved from preintervention, remained suboptimal (27.7%), and the direction of change varied by health facility, with a decline in one health center at the postintervention measurement. Similarly, the use of a sterilized delivery kit at endline, though improved from preintervention, was not universal for all babies born (65.7%). Challenges in sterilization of reusable medical equipment and running out of stock of clean delivery kits might have contributed to this outcome. Previous studies on essential newborn care practices have also shown varied levels of success. In the India BetterBirth Program, adherence to hand hygiene by the birth attendant was the lowest of the practices they measured and declined at the 12 -month follow-up in the intervention arm [32].

Predischarge care, including newborn assessment at 2 h postbirth, was not common practice and did not improve. Length of stay following childbirth is a critical time to monitor for danger signs and to educate the mother/caretakers on newborn care, danger signs of newborn illness, and breastfeeding. The limited space or capacity to admit at the health facilities, the desire by families to go home, and lack of prioritization might have contributed to the short length of stay and the suboptimal predischarge care and education provided to mothers. Future trainings might benefit from sessions that focus on the health workers’ skills in educating and counseling mothers and caretakers, checklists to ensure that routine interventions are provided, and readiness for discharge assessment of newborns.

Overall, essential newborn care practices at baseline were much lower than reported in other African countries [33]. One of the consequences of protracted conflict is the limited opportunity for continued education and skill-based trainings [30, 34]. Care practices that were not targeted or emphasized by the intervention package, including quality of antenatal care, did not improve from baseline. Acquisition of essential knowledge and skills for newborn care is critical to improving the care of newborns; however, to effect change on newborn survival, capacity-building approaches in humanitarian settings need to include the pregnancy continuum, maternal care, and care for small and sick newborns. Humanitarian settings, such as Somalia, often depend on prepackaged supplies or kits in the absence of a functioning national medical supply chain system [20]. In our study, medical supplies that needed sterilization were not preferred by providers, and supplies that need installation were not installed, limiting their applicability. Future medical kit provisions should include installation or training on installation and should be appropriate for the context.

The newborn register enabled health workers to record newborn data, promoted evidence-based practices such as recording birth weight in grams, and the use of data for programming. However, careful consideration should be given to avoid overwhelming health workers with multiple record systems and reports and duplicating efforts. The results of this study suggest that the Field Guide recommended essential newborn care practices, newborn kits, and the data system are feasible and have the potential to improve essential newborn care practices in the context of protracted humanitarian crises.

### Strengths and limitations

Interrupted time-series study design is increasingly used for the evaluation of public health interventions, and it is particularly well suited to interventions introduced over a clearly defined time-period. Our study had a clearly defined time for pre and postintervention measurements, and the interventions were introduced after the preintervention measurement was completed, decreasing the likelihood that concurrent changes in practice influenced the outcome. The 18-month follow-up enabled us to assess the retention of knowledge and skills. The use of observation rather than self-reported or register-based information was a strength of this study. However, the study had several limitations. First, we were unable to quantify individual (provider) levels of change in practice. Second, our estimates had wide confidence intervals overall, reflecting the small number of clusters in the study, leading to corrections widening the confidence intervals. This wide confidence interval is much more visible in the GEE model, where there were also small numbers present in the comparison groups. Third, we were unable to train all of the health workers at the four health centers. Fourth, we lost some mothers to follow-up at the postnatal visit, which might have affected the outcome. There is also the possibility of the Hawthorne effect, where providers might have performed differently under observation than in routine practice [35]. Fifth, the study was performed at four health facilities, and the implementation package and findings of this study might not be generalizable to national or large-scale initiatives. Finally, temporal changes in context or policy during the implementation period could have influenced the results.

## Conclusion

We found that the intervention package, which included training of providers, provision of newborn medical commodities, and the installation of a newborn register, was feasible and effective in improving essential newborn care practices at the primary health facility level. There were areas of essential newborn care such as handwashing and predischarge education that did not improve and that will require a different approach and/or more intensive training and supervision. Sustained knowledge and skill retention at the 18-month follow-up is a promising result for program sustainability. The results of this study could provide a foundation for policies such as the national Every Newborn Action Plan that is under development and the scale-up of essential newborn care programs in Somalia.

## Supplementary information

**Additional file 1.** Supplemental 1.

**Additional file 2.** Supplemental 2.

## Data Availability

All relevant data used and/or analyzed for the study are available in anonymized form from the corresponding author upon reasonable request.

## Abbreviations

CDC: U.S. Centers for Disease Control and Prevention
IDPs: Internally Displaced Persons
WHO: World Health Organization

## Footnotes

1 Exact count agreement divided by total count. 3600/3807
