# Supplementary material for: Effectiveness of clinical training on improving essential newborn care practices in Bossaso, Somalia: a pre and postintervention study
Source: BMC Pediatr. 2020 May 13;20:215. doi: 10.1186/s12887-020-02120-x (PMC7222459; doi:10.1186/s12887-020-02120-x)
Supplement: Supplementary file 1 — Additional file 1. Supplemental 1. [file 12887_2020_2120_MOESM1_ESM.pdf]

Supplemental 1

**Newborn Kit: Medical Supplies, Equipment's, and Drugs**	
1	blanket, baby, 50 x 75 cm, polyester fleece
2	blood glucose meter, range 40-600 mg/dl, LCD display 35 x 31.5 mm, 360 memory positions (BG-102)
3	blood lancets, sterile, disposable
4	blood glucose test strips (BS-101), for use with BG-102 glucose meter
5	cap, newborn
6	doppler apparatus, pockettype, LCD display, FHR display, interchangeable 2 Mhz probe (Sonoline B)
7	ultrasound gel, 250 ml
8	gloves, examination, latex, size medium, pre-powdered, disposable, non-sterile
9	light, examination, floortype, flexible neck, 220V lamp (bulb not included), on castors
11	suction device, penguin-model, silicone, autoclavable (Laerdal 986000)
12	needle, hypodermic, Luer, 25 G x 5/8" (0.50 x 16 mm), sterile, disposable (orange)
13	respiration rate timer, for ARI programmes, with batteries (S
14	resuscitation bag, with mask no. 0 and 1 (Laerdal NeoNatalie Resuscitator 846030)
15	scalp vein infusion set 25 G (0.50 mm), sterile, disposable
16	syringe, hypodermic, Luer, 3-part, 1 ml, sterile, disposable
17	table for baby reanimation with overhead heater, mattress, skin/air temperature control, trolley (RHW2102A)
18	thermometer, electronic, digital display (Celsius)
19	towel, 60 x 80 cm, cotton
20	urine teststrips, 10 detections
21	RPR slide-test, 100 tests (keep cool 2-8ºC)
22	weighing scale, baby, electronic, digital, 20 kg, graduation 5 g
24	ampicillin 500 mg
25	cefotaxime sodium 1 g
26	ceftriaxone 250 mg
27	gentamycin sulphate 40 mg/ml, 2 ml
28	vitamin K-1 (phytomenadione) 1 mg/ml, 1 ml (oily)
29	kit, emergency reproductive health, clean delivery, A
30	kit, emergency reproductive health, clinical delivery assistance, reusable equipment
31	kit, emergency reproductive health, clinical delivery assistance, drugs and disposable equipment)
32	kit, emergency reproductive health, vacuum extraction delivery (HM Healthcare model)
