# Supplementary material for: Effectiveness of clinical training on improving essential newborn care practices in Bossaso, Somalia: a pre and postintervention study
Source: BMC Pediatr. 2020 May 13;20:215. doi: 10.1186/s12887-020-02120-x (PMC7222459; doi:10.1186/s12887-020-02120-x)
Supplement: Supplementary file 2 — Additional file 2. Supplemental 2. [file 12887_2020_2120_MOESM2_ESM.pdf]

**Tilmaamaha:**<br>Buuxi diiwaanka hal-layn cunuggiiba. Jawaabaha macquulka ah ee kolom kasta waxay ku taxan yihiin hoos. Haddii dhibaatooyin jiraan, fadlan kasoo bixi hal-jawaab - haddii cunugga ama hooyadu ay leeyihiin dhibaatooyin fara-badan, qor dhibaatada ugu halista badan.<br><br>Haddii mataano yihiin: mataan kasta waa in laga-buuxiyaa laynkiisa diiwaanka. Kolomka saddexaad, calaamadi Maxsuulka mataankaas (tusaale ahaan, mid ka-mid ah mataanaha wuxuu noqon kara ''mataan nolol ku dhashay'' kan-kalena wuxuu noqon karaa ''mataan dhicis ah''). Kaliya buuxi dhibaatooyinka hooyada iyo natiijada hooyada hal-mar (waxaad ku-qori kartaa ''mataano'' kolomka dhibaatooyinka hooyada, mataanka kalena ku-qor kolomka natiijada hooyada).	**Instructions:**<br>Fill out the register with one line for each baby. The possible responses to each column are listed at the bottom. For complications, please only pick one response- if the baby or mother has multiple complications, put down the most serious complication.<br><br>For twins: each twin should have its own line in the register. For the third column, mark the outcome for that specific twin (for example, one twin could be “twin livebirth” and the other could be “twin stillbirth”). Only fill out the maternal complication and maternal outcome once (you can write “twin” in the maternal complication and outcome columns for the second twin).

**Qeexidda. Dhibaatooyinka Murjuca**		**Definitions. Newborn Complications**	
**Jirrooyin muuqda**	Ma-lahan dhaqdhaqaaq<br>Miyir la' (aan miyir lahayn)<br>Soo-maray gariir (lahaan jiray gariir)<br>Waxba Nuugayn<br>Dhiig-bax aad u-daran<br>Nasista oo soo-buuran	**Critical Illness**	No movement<br>Unconscious<br>History of convulsions<br>Unable to feed<br>Severe bleeding<br>Bulging fontanelle
**Ikfashin / Caabuq aad u-daran**	Qando (heer-kul ka badan ama le'eg 38 darajo)<br>Heer-kulka jirka oo yaraada (heer-kul ka-yar 35 darajo)<br>Nuugidda ama cabbidda murjuca oo xun<br>Dhaq-dhaqaaq yaraaday<br>Feeraha oo qaad-qaadma (neef-gur)	**Severe Infection**	Fever (temperature greater than or equal to 38 degrees Celsius)<br>Hypothermia (temperature less than 35.5 degrees Celsius)<br>Poor feeding<br>Reduced movement<br>Severe chest in-drawing
**Neefsi degdeg (neef-gur)**	Tirada Neefsiga oo ka-wayn 60 neefsi daqiiqaddiiba	**Isolated Fast Breathing**	Respiratory rate > 60 breaths per minute
**Ku-dhashay naqaskoo ku dhaggan**	Murjucu ma-bilaabin neefsi dabiici ah	**Birth Asphyxia**	Newborn does not start spontaneous breathing
**Dhicis**	Ilmo ku-dhashay ka-hor intaanu dhammaysan 37 asbuuc	**Prematurity**	Babies born before completed 37 weeks of gestation
**Cagaarshow**	Midabka maqaarka iyo indhaha oo jaalle isu baddala.	**Jaundice**	Yellowish discoloration of the skin and the whites of the eyes

**Qeexidda: Dhibaatooyinka Hooyada**		**Definitions: Maternal Complications**	
**Dhiig-bax Dhalmada ka-dib**	Waxaa lagu qeexaa marka hooyadu lumiso wax ka-badan 500 ml ama 1,000 ml oo dhiig ah inta lagu jiro 24ka saac ee ugu horreeya dhalmada ka-dib.	**Postpartum Hemorrhage (Bleeding)**	Defined as the loss of more than 500 ml or 1,000 ml of blood within the first 24 hours following childbirth
**Foosha oo dheeraata**	Waa Xaalado badan oo ay ka-mid yihiin foosha oo qaadata wax ka-badan 12 saacadood, miskaha hooyada iyo uurjiifka oon is le-kayn ama soo beegnaanta cunugga oo aan caadi ahayn	**Obstructed/ Prolonged Labour**	Broad range of conditions including labour lasting more than 12 hours, feto-pelvic disproportion or abnormal fetal presentation
**Bare-eclampsia/ Eklamsia**	Bare-eclampsia/ Eklamsia waa mushkilad uurka la xiriirta oo lagu garto Cadaadiska Dhiiga oo sarreeya. Calaamadaha kale waxaa ka-mid ah Barar iyo kaadida oo borotiin soo raaco.	**Pre-eclampsia/ eclampsia**	Preeclampsia is a complication of pregnancy that is associated with high blood pressure. Other signs and symptoms include swelling (edema) and protein in the urine.
**Ikfashin aad u-daran/ Jirrooyin aad u-daran**	Qandho/Xummad: (Heer-kulka jirka oo ka-sarreeya 37.5 darajo), Qaybta hoose ee caloosha oo aad u-xanuunta, Dhiigga ka-yimaada xubinta taranka oo ura ama siifad xun yeesha	**Severe Infection/ Severe illness**	Fever (temperature >37.5 degree centigrade), severe lower abdominal pain, foul smelling vaginal discharge

Newborn Register

Register # / Lambarka Diiwaanka	Date of Birth / Taariikhda dhalashada	Birth Outcome / Maxsuulka dhalashada	Birth weight / Culayska Dhalashada	Gestational Age in Weeks LMP / Sadka-uurka oo asbuuc ah	Mode of Delivery / Qaabka dhalmada	Time of Birth / Waqtiga dhalmada	Gender of Newborn / Jinsiga murjuca	Newborn Resuscitated? Dib u-neefsiinta murjuca?	Outcome of Newborn Resuscitation / Maxsuulka Dib u-neefsiinta murjuca	Newborn Complications / Dhibaatooyinka murjuca	Newborn Outcome / Maxsuulka Dib u-neefsiinta	Maternal Complication / Dhibaatooyinka hooyada	Maternal Outcome / Maxsuulka Hooyada	Time of Discharge, Referral, or Death / Waqtiga bixida, gudbin, Murjuc Dhintay
Match delivery register number / Ka tixraac lambarka diiwaanka hooyada dhashay	DD/MM/YYYY<br>MM/BB/SSSS	Livebirth (single), Livebirth (Twins), Livebirth (one twin), Stillbirth (single), Stillbirth (twins)<br>Hal murjuc oo nolol ku dhashay, Mataano Nolol ku-dhashay, Hal cunug mayd ah, Mataano: mid nool yahay midna mayd yahay, Mataano mayd ah	in grams<br>oo garaam ah (u-dhexeeya: 1000 - 5000)	Sadka-uurka asbuuc ahaan oo lagasoo bilaabo xilligii ugu danbaysay caadada [28 asbuucs – 43 asbuuc]	SVD / AVD<br>Caadi ama dabiici u-dhashay / Lagu dhaliyey qalab (Vacuum)	HH:MM<br>SS:DD	Female / Male<br>Dhadig / Lab	Yes / No<br>Haa / Maya	Survived / Death / Survived and Referred<br>Badbaaday / Murjuc Dhintay / Badbaaday oo la-gudbiyey	Critical Illness, Severe Infection, Isolated Fast Breathing, Neonatal Tetanus, Birth Asphyxia, Congenital Abnormality, Prematurity, Jaundice, Other, None<br>Jirro muuqata, Ikfashin/Caabuq aad u-daran, Neefsi degdeg (neef-gur), Teetanada murjuca, Ku-dhashay naqaskoo ku dhaggan, Ku-dhashay qaab-darro, Dhicis, Cagaarshow, Wax-kale, Midna	Discharged (Recovered), Referred, Discharged Against Medical Advice, Newborn Death<br>Ka-baxay xarunta (Bogsaday), La-gudbiyey, Ka-baxay xarunta (ka-hor yimid talooyinka caafimaadka), Dhimasho Murjuc	Bleeding, Obstructed/ prolonged labor, Pre-eclampsia/ eclampsia, Severe Infection, Other, None<br>Dhiig-bax, Foosha oo dheeraata, Eklamsia, Ikfashin/ Caabuq aad u-daran, Wax-kale, Midna	Discharged (recovered), Referred, Discharged Against Medical Advice, Maternal Death<br>Ka-baxay xarunta (Bogsaday), La-gudbiyey, Ka-baxay xarunta (ka-hor yimid talooyinka), Dhimasho hooyo	HH:MM<br>SS:DD
